# Associations of sexual behavioral aspects and sexual scripts among college students: A cross-sectional study

**DOI:** 10.18502/ijrm.v20i11.12358

**Published:** 2022-12-10

**Authors:** Arezo Omidvar, Malek Mirhashemi, Habib Yousefi, Effat Merghati Khoei

**Affiliations:** ^1^School of Psychology, Islamic Azad University, Roudhen Branch, Tehran, Iran.; ^2^The Iranian National Center for Addiction Studies (INCAS), Tehran University of Medical Sciences, Tehran, Iran.; ^3^The Family and Sexual Health Division, Brain and Spinal Cord Injury Research Center (BASIR), Neuroscience Institution, Tehran University of Medical Sciences, Tehran, Iran.

**Keywords:** Sexual scripts, Sexual behavior, Sexuality, College student, Iran.

## Abstract

**Background:**

Sexual scripts (SSs) are formed based on the gendered culture in societies.

**Objective:**

To evaluate the associations between the sexual behavioral aspects: capacity, motivation, performance, and SS amongst Iranian college students.

**Materials and Methods:**

From September to December 2020, we recruited 400 college students who were married, had no acute or chronic diseases, was no drug abuser, and were not pregnant at the time of the study. The demographic questionnaire, sexual behavior assessment, and SSs questionnaires were completed by all the participants. Descriptive statistics and inferential statistics, Pearson correlation coefficient, and multivariate regression were used for data analysis. We also employed an independent *t *test to compare sexual behavior-related variables: sexual capacity, sexual performance, sexual motivation, and SSs in 2 female and male student groups.

**Results:**

Men and women were significantly different in the component of sexual behaviors. Women had higher sexual motivation than men. The participants' SSs were positively correlated with all 3 components of sexual behaviors at a significant level of p ≤ 0.001. Of demographic characteristics, only women's age had a significant correlation with sexual capacity. For men, education level and economic status were positively correlated with all components of men's sexual behaviors.

**Conclusion:**

Sexual behaviors seem to be highly gendered, particularly, in the motivational component. Based on the results, SSs are more determinant than demographics. We recommend gender-sensitive and cultural-oriented sexuality educations for Iranians to provide fundamental changes in the SSs.

## 1. Introduction

Sexuality is part of human integrity. It encompasses discourses between systems in which sexuality is created, sustained, and interpreted. Thus, sexuality is not a “spontaneous” phenomenon arising from innate biological drives. It is influenced by biological, cultural, ethical, legal, historical and religious, and spiritual factors (1). In line with our argument, sexual script theory also highlights that sexual behaviors follow various scripts (2). In a survey, data collected from students (n = 614) on 2 campuses revealed common types of hooking up and sexual relationships. The researchers concluded that gender, race, alcohol consumption, and perceptions of sexual interactions have an impact on their sexual relationships. Whereas, social class, Greek affiliation, religiosity, religious attendance, or perceptions of campus norms and sexual interactions revealed no significant impact on the subjects' romantic and sexual relationships (3).

Theory and research emphasize differences in men's and women's sexual behavior, including sexual capacity, motivation, and performance (4-8). No question that “scripts are involved in learning the meaning of internal states, organizing the sequences of specific sexual acts, decoding novel situations, setting the limits on sexual responses, and linking meanings from nonsexual aspects of life to specifically sexual experiences” (3, pp.19). According to sexual scripting theory, people's sexual behaviors, feelings, and thoughts are scripted in the given social context (9). Inspiring the sexual script theory, sexual behaviors are not only defined as erotic or genital-based, but they also involve gender roles, reproduction, and life-enhancing behaviors (10). From Hilbert's standpoint, 3 main factors construct one's sexual behaviors: sexual capacity (i.e., what a person can do), sexual motivation (i.e., what a person wants to do); and sexual performance (i.e., what a person does), which all alter individuals' sexual behaviors (11).

Similar to others, in Iranian culture, sexual behaviors are markedly different for men and women. In this culture gender issues and sexuality are evidently constructed in a typical patriarchal society. In Iran, religious teachings tie strongly to Iranians' understanding of sexuality, and premarital or extramarital sexual relationships are forbidden for both men and women (2). According to Muslim scholars, the subject of women and sexuality has long been controversial and has remained a topic of debate among scholars (12, 13). According to a national survey classifying sexual scripts (SSs) (traditional, relational, or recreational); respondents reported that their religiosity and the value system have profound impact on their sexual attitudes (14).

Therefore, in the field of sexology, identifying gender differences in any society seems necessary. In this study, our goal was to identify Iranian college students' SSs based on gendered culture. It is femininity and masculinity that formulate sexual behavior and warranted its quality. Our findings would enable us to design and implement culturally sensitive interventions to prevent harm or promote Iranians' sexual life quality. In this article, we report associations of sexual behavioral aspects and SSs among college students. Also, the associations between the aspects of sexual behaviors and the demographics of the students (men and women) have been assessed.

## 2. Materials and Methods

### Setting and participants

In this cross-sectional study, 400 married and full-time college students (men and women) from the Roudehen branch, Islamic Azad University, Tehran, Iran were recruited from September to December 2020. All students with acute or chronic diseases, drug abuse, pregnancy, psychological disorder under treatment, and self-reported marital conflicts at the time of data collection were excluded. Using flyers and announcements, the participants were invited to participate in the study and asked to fill out the demographic form, sexual behaviors assessment questionnaire, and the SSs questionnaire.

### Outcome measures

The participants were compared in terms of age, gender, marital and economic status, education, number of children, and medical history, also as sexual behavior-related variables including sexual capacity, performance, motivation, and SSs in 2 female and male student groups.

Sexual behaviors assessment questionnaire is a 33-item questionnaire developed and validated by Ghorashi et al. (2). Its Cronbach's alpha coefficient was previously demonstrated for women (0.81) and 29-item with an alpha coefficient of 0.62 for men. This questionnaire has been designed in the 5-point Likert spectrum which ranges from one “not at all” to 5 “too much”. It includes the 4 subscales of sexual performance, capacity, motivation, and SSs as “positive response to the wife/husband's sexual demands will bring reward in the hereafter”; “a positive response to the wife/husband's sexual demands makes the spouses' relationship more intimate and creates peace in the family”; “a positive response to the wife/husband's sexual demands prevents the spouse from being attracted to the others”.

### Ethical considerations

The research proposal was approved by the Ethics Committee of Tehran University of Medical Sciences, Tehran, Iran (Code: IR.TUMS.VCR.REC.98-3-146-46145). Students participated in the study voluntarily, and oral and written consent was obtained from all participants before entering the study. Participants were also assured that their information was confidential.

### Statistical analysis

We first hypothesized SSs would (a) relate to the men and women's sexual behaviors (capacity, motivation, and performance), (b) sexual behaviors relate to their demographic characteristics, and (c) the SSs' correlation with sexual behaviors would be stronger in women than men, as Iranian women seem to be significantly more restricted in sex than men. Using Statistical Package for the Social Sciences (SPSS) version 25 headquartered in Chicago and incorporated in Delaware, indicators of central tendency, dispersion, and distribution of scores were used to describe the variables. In the statistical analysis, due to the distanced-based nature of the measurement scale, and the research hypotheses, Pearson correlation coefficient, and multiple regression analysis were utilized. We employed an independent *t* test to compare women and men in terms of sexual behavior-related variables and SSs. A significant level in the study was considered < 0.05.

## 3. Results

During the announcement period, 440 people volunteered to participate in the study, of which 23 were not eligible to enter and 17 were excluded from the analysis due to unfinished questionnaires. The indicators of mean, standard deviation, and percentage of participants are shown in table I. We employed an independent *t *test to compare the study participants in 2 groups of women and men in terms of sexual behavior-related variables including sexual capacity, performance, motivation, and SSs (Table II). Men and women were significantly different from each other only in the component of sexual motivation (p = 0.05). According to the mean values of these 2 groups, it was revealed that women have higher sexual motivation than men in this study. The square of multiple correlations (R2 = 0.128) showed that demographic variables predict 12.8% of the variance of sexual capacity in women. Sexual script significantly predicted 27.5% of the variance of sexual capacity in women (R2 = 0.275) (Table III). Similar to women, demographic variables predict 26.1% of the variance of sexual performance in men (R2 = 0.261). Sexual scripts significantly predicted 31.2% of the variance of sexual performance in men (R2 = 0.312) (Table III). This means that the inclusion of SSs in the prediction equation explains 31.2% of the variance in male sexual performance. With the inclusion of SSs in the prediction equation and by controlling the effect of demographic variables, the variance in a man's sexual capacity was revealed higher by 2%.

**Table 1:** Demographic characteristics of study participants in 2 groups (n = 200/each)


**Variables**		**Female students**	**Male students**	**P-value**
**Age (yr)**		37.87 ± 7.15	32.55 ± 6.45	< 0.001
**Spouse's age (yr)**		41.37 ± 8.72	36.34 ± 6.06	0.11
Education level*				
	**Diploma**	5	46	
	**Associate Degree**	7	0	
	**Bachelor**	143	108	0.25
	**Master**	40	46	
	**Ph.D.**	5	0	
Spouse's education*				
	**Diploma**	36	33	
	**Associate Degree**	17	0	
	**Bachelor **	89	124	< 0.001
	**Master**	44	43	
	**Ph.D.**	14	0	
Economic status**		6.13 ± 1.73	5.15 ± 1.76	< 0.001
Data presented as Mean ± SD for continues and frequency for categorical variables, *Last educational certificate, **Too bad/not too bad/fairly good/very good				

**Table 2:** Comparison of sexual behaviors (3 components) and SSs in 2 groups of men (n = 200) and women (n = 200) students


**Variables**		**Mean**	**df**	***t***	**P-value**
**Sexual capacity**					
	**Female students**	24.83 ± 6.16		
	**Male students**	25.85 ± 7.47	382	-1.46	< 0.001
**Sexual performance**					
	**Female students**	21.13 ± 8.21		
	**Male students **	20.44 ± 7.71	369	0.82	< 0.001
**Sexual motivation**					
	**Female students**	20.60 ± 9.92		
	**Male students**	18.44 ± 7.02	373	2.40	< 0.001
**Sexual script**					
	**Female students**	37.13 ± 9.52		
	**Male students**	36.66 ± 8.00	369	0.50	0.09
Independent *t* test to compare the study participants in 2 groups of women and men, df (degrees of freedom), *t* (the *t* test statistic)					

**Table 3:** Multiple regression analysis in predicting women's sexual capacity (n = 200) and men sexual performance (n = 200)


**Demographic variables**		**B**	**SE**	**β**	**t**	**P-value**
**Women's sexual capacity**						
	**Age (yr)**	-0.283	0.069	-0.254	-4.07	< 0.001
	**Education**	0.938	0.813	0.075	1.15	0.25
	**Spouse education**	-0.466	0.451	-0.069	-1.03	0.30
	**Economic status ${}^{\text{a}}$ **	0.852	0.286	0.185	2.98	< 0.001
	**Sexual capacity ${}^{\text{b}}$ **	1.060	0.286	0.185	6.27	0.01
**Men sexual function**						
	**Age (yr)**	-0.021	0.048	-0.026	-0.43	0.67
	**Education**	1.500	0.564	0.197	2.66	< 0.001
	**Spouse education**	-0.208	0.597	-0.025	-0.35	0.72
	**Economic status ${}^{\text{c}}$ **	1.014	0.204	0.340	4.98	< 0.001
	**Sexual performance ${}^{\text{d}}$ **	0.495	0.131	0.340	3.79	< 0.001
${}^{\text{a}}$ R^2^ = 0.147, ${}^{\text{b}}$ R^2^ = 0.275, ${}^{\text{c}}$ R^2^ = 0.261, ${}^{\text{d}}$ R^2^ = 0.261, B (the unstandardized beta), SE (standard error), β (the standardized beta), t (T Ratio)						

## 4. Discussion

In this study, we found men and women different from each other except for the component of sexual motivation. According to the mean values of the 2 groups, women showed higher sexual motives than male students. Women's SSs revealed a positive correlation with all 3 components of sexual behaviors similar to their male counterparts. Of demographic characteristics, only women's age revealed a significant correlation with sexual capacity compared with their male counterparts whose education level and economic status showed a positive correlation with all components of their sexual behaviors.

Compared to others, our results show that age (negatively) can significantly predict sexual capacity in women (3). Other research showed that marital satisfaction was significantly related to the level of love, interest, socioeconomic status, and education of couples (4). Among the demographic variables, only the level of education and economic situation positively and significantly predicts male sexual performance, which is in line with other reports (5).

We also found a strong correlation between the dimensions of sexual behavior and SSs in Iranian men and women, during the study. Our findings regarding sexual differences between men and women in sexual behaviors and SSs show that women, compared to men, have a superior status in sexual capacity, sexual motivation, and SSs. In contrast, men are superior in sexual function compared to women. In this study we found that SSs (positively) can predict sexual capacity, motivation, and function. In Iran, sexuality is highly gendered based on different roles labeled as femininity or masculinity (9). A study with Iranian women concluded that SSs determine the means participants experience their sexual interactions (13). In her research report, Merghati Khoei argued that marital life is mainly integrated with its sexually-related concepts in the Iranian culture. However, majority of women who live in this culture lack appropriate language to express their sexuality. She points out that Iranians believe that talking about their marital lives in public will dishonor them from the society's lenses (1). This finding is consistent with the research (6) on women of menopausal age and other men and women with various sexual orientations (7). Several studies reported gender as a significant moderating factor in sexual enjoyment (8).

It was reported that the role of intimacy between couples in promoting sexual motivation, following that, increases the sexual capacity and functioning of women throughout aging (3). Similarly, there is a negative and significant relationship between primary maladaptive schemas and marital adaptability in men and women (9). A positive and significant relationship between the total score of primary maladaptive schemas and the score of fear of intimacy for men and women was reported (10, 11). Findings related to path analysis also showed that a fear of intimacy mediates the relationship between primary maladaptive schemas and marital adaptability. Inspiring the actor-partner interdependence model, a research report indicated that sexual motives medicate the association between attachment orientation and sexual satisfaction as well as the functioning of couples. Men's sexual satisfaction, sexual intimacy, and orgasmic response are warranted when they actor roles compared with their partners (12). Based on the current study findings, it can be concluded that primary maladaptive gender schemas play a major role in marital maladaptation through fear of intimacy. In a similar study on men and women in Tehran, the direct effect of schemas on the style of loving and couple intimacy at the level (p < 0.05) was investigated (13). In another study, married women in Mobarakeh city found that the relationship between sexual self-concept and sexual function was significant. Positive self-conceptual variables, direct and negative sexual self-concept variables inversely were predictors of women's sexual function (14). We found that SSs significantly predict sexual capacity, motivation, and functioning in men. In particular, there are 3 styles of working with SSs: (I) conformity, in which personal SSs overlap with traditional scripts for sexual behavior; (II) exceptions, in which people accept a sexuality scenario at the cultural level as reality, but create exceptional rules for themselves; and (III) transformation, in which people either try to reconstruct sexuality scripts at the cultural level or interpret their unusual styles as normative. Changing SSs could potentially lead to reduced sexual inequality in the sexual realm and increase the opportunity for sexual satisfaction, safety, and well-being, especially for women (15, 16).

In response to the research question about whether SSs are different in men and women, other scholars believe that women consider sexual intimacy as a precondition of sexual interests (17), while based on notions of hegemonic masculinity and sex as a drive, men expect to achieve sexual intimacy through sexual interactions (16). It would not be surprising if women experience low-sexual interest or poor functioning, as the quality of interpersonal relationships reveals poor status. In line with our findings, in a study, the relationship between sexual behavior and sexual schemas in men and women was measured and it was concluded that women are significantly different from men in specific areas such as orgasm and feelings of resentment and resentment of the husband. They have also stated that gender role schema in women and men affect their satisfaction as well as secure sexual behaviors (18). In line with others, we do highlight the complex relationship between religiosity and sexuality (19, 20) and argue that SSs act as a core religious principle in line with Islamic principles and values explaining the belief that unconditional sexual submission will bring reward in the hereafter and is a form of devoted worship (21).

For Iranians, the marital institution is the only context where men and women experience sexual encounters believing that sexual interaction warrants marital intimacy and longevity. With such a script, it is not surprising that our participants showed sexual submission to the partner's sexual demands as the benchmark of a more intimate and peaceful partnership. We were not surprised to find that participants used their sexual potential as a preventive instrument against marital infidelity. A strong belief explains this script among Iranians that if a person does not respond to their partner's sexual needs; they may be involved in a wedlock affair and ruin the family's sexual value system.

### Strengths and limitations

Real-life data associated with experiential outcomes are not always real-valued. In other words, opinions, perceptions, ratings, etc., are often identified as vague in nature, especially when they come from human evaluations. To overcome this drawback, we used a standard Iranian questionnaire to assess sexual behaviors that had characteristics, sensitivity, and cultural compatibility that facilitated participants' responsiveness. Cronbach's alpha coefficients of sexual motivation and SSs were slightly found low in men. Therefore, we need to be careful in interpreting the findings related to the male participants. There is a significant difference between the 2 groups of men and women. A possible explanation for this difference can be the measure we used for men (29-item) and women (33-item). This study only used a quantitative approach, while a qualitative approach could suggest optimal findings in parallel.

## 5. Conclusion 

According to the findings of the present study, it seems that the sexual behaviors of men and women are strongly influenced by their SSs than by their demographic characteristics. In light of the conclusion, it is recommended that culturally sensitive sexual training programs be implemented in the country to create fundamental upbringing and guidance in the adjustment of scripts. Also, premarital education programs need to be designed to correct negative attitudes toward sexuality and make positive changes in critical SSs. Furthermore, professionals working in health and sexuality need to be sensitive to their clients SSs.

##  Conflict of Interest

No conflict of interest in this study.
